# Integrated Framework of Load Monitoring by a Combination of Smartphone Applications, Wearables and Point-of-Care Testing Provides Feedback that Allows Individual Responsive Adjustments to Activities of Daily Living

**DOI:** 10.3390/s18051632

**Published:** 2018-05-19

**Authors:** Peter Düking, Silvia Achtzehn, Hans-Christer Holmberg, Billy Sperlich

**Affiliations:** 1 Integrative and Experimental Exercise Science & Training, Institute for Sport Sciences, University of Würzburg, 97082 Würzburg, Germany; billy.sperlich@uni-wuerzburg.de; 2 Institute of Cardiology and Sports Medicine, German Sport University, 50933 Cologne, Germany; achtzehn@dshs-koeln.de; 3 The German Research Centre of Elite Sport, German Sport University, 50933 Cologne, Germany; 4 School of Sport Sciences, UiT The Arctic University of Norway, 9019 Tromso, Norway; Hans-Christer.Holmberg@miun.se; 5 Swedish Winter Sports Research Centre, Department of Health Sciences, Mid Sweden University, SE-831 25 Östersund, Sweden

**Keywords:** biofeedback, eHealth, individualized training, injury prevention, IoT, load management, periodization

## Abstract

Athletes schedule their training and recovery in periods, often utilizing a pre-defined strategy. To avoid underperformance and/or compromised health, the external load during training should take into account the individual’s physiological and perceptual responses. No single variable provides an adequate basis for planning, but continuous monitoring of a combination of several indicators of internal and external load during training, recovery and off-training as well may allow individual responsive adjustments of a training program in an effective manner. From a practical perspective, including that of coaches, monitoring of potential changes in health and performance should ideally be valid, reliable and sensitive, as well as time-efficient, easily applicable, non-fatiguing and as non-invasive as possible. Accordingly, smartphone applications, wearable sensors and point-of-care testing appear to offer a suitable monitoring framework allowing responsive adjustments to exercise prescription. Here, we outline 24-h monitoring of selected parameters by these technologies that (i) allows responsive adjustments of exercise programs, (ii) enhances performance and/or (iii) reduces the risk for overuse, injury and/or illness.

## 1. Introduction

To optimize performance, athletes schedule their training and recovery in periods, i.e., in micro- (e.g., within a single session of training or from day-to-day) or macro-cycles (e.g., on a weekly or monthly basis), with variations in intensity, volume and/or frequency.

Adaptation (e.g., with respect to elevated power output or oxygen uptake) to standardized training varies considerably between individuals [1,2]. Rigid adherence to a standardized or pre-defined program of exercise, without routine monitoring of physiological and perceptual responses and appropriate responsive adjustments, may result in underperformance and/or compromise health [3]. The training stimulus becomes inappropriate when the external load (defined here as the work/physical activity completed) is unsuited to the psycho–physiological responses of the individual involved (referred to here as the internal load) [4,5].

Monitoring load is extremely complex, since all of an individual’s systems adapt to numerous simultaneous stimuli in an integrated manner. It has been proposed that 24-h monitoring might help take into account the various factors that influence overall adaptation to exercise, thereby improving our insight into the interdependencies in this context between the stress of training, recovery, off-training activities of daily-life, and various other stimuli (e.g., temperature, humidity, psycho-social stressors, and many more) [6] and may allow individual responsive adjustments to exercise programming. From a practical perspective, including that of coaches, monitoring of load designed to detect potential changes in health and performance should be valid, reliable and sensitive, as well as time-efficient, easily applicable, non-fatiguing and as non-invasive as possible [7].

In this context, smartphone applications (Apps), wearable sensors (Wearables) and point-of-care-testing (POCT) all allow (i) high-resolution and/or regular monitoring of a variety of relevant psycho–physiological markers of internal and external load; (ii) minimally or non-invasive collection of data; (iii) rapid evaluation of this data and, thereby, potentially instant (bio-)feedback; (iv) measurements in a variety of different settings (e.g., at home, while training, during competition, while traveling, during daily-living); and/or (v) monitoring without the involvement of sophisticated medical personnel or the necessity for a laboratory [8,9,10].

Interestingly, despite these considerable advantages, there appears to be little awareness of the capabilities of Apps, Wearables and POCT to provide integrated and instant feedback to athletes and coaches that allows adjustment of exercise to minimize risks to health and optimize adaptation. Accordingly, the present aim was to describe certain approaches of this nature that might be effective.

## 2. Monitoring Parameters of External and Internal Load

The various parameters associated with external and internal training load all appear to be of potential interest in connection with monitoring responses [4,8,11]. Here, we focus on external parameters which describe the workload completed by an individual and internal psycho-physiological indicators which can assist coaches in modifying the external load in an appropriate manner. We have focused on load parameters currently monitored by Apps, Wearables, and/or POCT devices by minimally or non-invasive sampling of capillary blood or saliva, since such sampling does not require trained medical personnel. Although certain of their characteristics do overlap, we define Apps as executable software running on a handheld device such as smartphones and, sometimes, smartwatches; Wearables as lightweight devices worn close to, on or in the body that monitor, transmit and/or analyse data, providing bio-feedback [8], while POCT devices allow rapid biochemical, haematological, coagulation or molecular diagnostics at the point-of-care (e.g., the training facility), often in a minimally invasive manner [9].

It is beyond the present scope to consider all possible parameters and those we have chosen to focus on here are listed in Table 1 (external parameters) and Table 2 (internal parameters). While we motivate these choices, we are certainly aware that future technological advancements may well open more sophisticated perspectives.

## 3. Monitoring External Parameters

### 3.1. The Duration and Frequency of Training Sessions

The duration and frequency of exercise sessions, important and simple indicators of external load, can be easily monitored by (sport) watches. Many manufacturers provide the possibility to store this data automatically in a (cloud-based) database, which makes collection, aggregation and visualisation simple and straightforward.

### 3.2. Distance Covered

For many athletes the distance covered and time spent in different speed zones (expressed either in absolute or relative terms or as ratios, i.e., the acute/chronic workload ratio = the ratio of the workload during a single week to the average workload during a period of approximately four weeks) allow quantification of the external load and the distance covered exhibits a positive correlation to the likelihood of injury [12,13,14]. Relatively comfortable Wearable receiver units and Apps assess distance-related parameters employing global navigation satellite systems (GNSS) or local positioning systems (LPS).

### 3.3. Short Explosive Activities

Short explosive activities, such as movements involving a change in direction [15], tackling [16], sprinting [17] or throwing [18], may be utilized as measures of external load. For this purpose, three-dimensional accelerometers and gyroscopes that can be incorporated into various devices monitor parameters of body acceleration that can then be expressed in absolute or relative accumulated terms (44,40,45). For example, since the performance of numerous throws or tackling manoeuvres elevates the risk for injury [16,18,19], short explosive activities should be monitored closely.

### 3.4. Environmental Factors

A variety of environmental factors, including altitude, inclination, slope, temperature, exposure to ultra-violet radiation and humidity [20,21], can all exert a significant impact on external loading. These factors are readily monitored by sensors in Wearables.

### 3.5. Sleep

Developing research regarding sleep in athletes [22] reveals that sleep, performance and/or health are interconnected [23], as would be expected. The length of sleep and its relationship to the circadian rhythm can be estimated from the data supplied by Apps and Wearables employing various procedures [8,24,25].

### 3.6. Physical Activity Off-Training

Periods of off-training are often scheduled in a manner designed to optimize recovery and it is generally accepted that the type of activity (e.g., passive versus active) engaged in after exercise influences this recovery [26] and is therefore important to monitor [27]. Apps [28] and/or Wearables [29] can monitor off-training physical activity with, e.g., accelerometers and/or GPS-receivers and/or by photoplethysmography. Our knowledge concerning how off-training activities affect performance and/or health is presently seriously limited and needs to be extended.

## 4. Monitoring Internal Load

### 4.1. Parameters of General Health

Absence of illness and injury are obviously essential for athletic success. Several (sophisticated) parameters that reflect an athlete’s general health, level of stress and immunological status can all be assessed by, Wearables and/or POCT in a minimally or non-invasive manner. For instance, Wearables detect skin and body temperature at rest and during exercise, e.g., to assess heat-induced fatigue, and/or illness, as well as fever [30,31]. Various POCT devices can monitor such health-related variables as the white blood cell count (WBC; including determination of sub-populations and indicative of potential inflection) [32], high-sensitive C-reactive protein (hs-CRP, a marker of inflammation) and salivary immunoglobulin A (SIgA) (an indicator of mucosal immunity) [33]. In addition, POCT can detect toxic reactive oxygen species (ROS) produced during inflammation or exercise [34].

The blood level of haemoglobin, a crucial determinant of oxygen delivery, is influenced by the availability of iron and, thus, by the iron-storage protein ferritin. Prolonged and intense exercise is well known to stimulate rapid turn-over of erythrocytes, thereby causing loss of ferritin and a consequent reduction in the concentration of haemoglobin [35,36]. Monitoring of ferritin by POCT provides information about the transport of oxygen by the blood, allowing detection, e.g., of premature exhaustion. Furthermore, low levels of haemoglobin may reflect anaemia, whereas elevated levels may be indicative of dehydration.

### 4.2. Parameters Related to Cardiac Dynamics and Stress

With heart rate as a basis, Wearables can provide information on different parameters related to cardiac dynamics and stress [8,37]. Heart rate during exercise (expressed relative to an individual’s maximum) is often employed to quantify the intensity of exercise and can be used to monitor aerobic adaptation [37]. Variability in the heart rate (defined as the time that elapses between two consecutive R-R intervals) provides insight into the innervation of the heart by the autonomous nervous system [37,38,39] and appears to be relevant to chronic stress [40]. Such variability can be monitored by Wearables using different technologies [8], as long as potential confounding factors are carefully controlled for [37,41]. Heart rate recovery might indicate overreaching in athletes [42]. In addition, markers of potential myocardial stress, such as troponin and fatty-acid-binding protein (FABP), can be analysed by POCT [43].

### 4.3. Parameters Related to Bio-Psychological Stress

Elevated levels of salivary cortisol and alpha-amylase, both of which can be monitored readily by POCT, are indicative of internal stress [44,45]. This cortisol level increases in response to intense physical exercise. Elevated levels of cortisol, which is considered to be the hormone primarily responsible for catabolic processes, can augment protein degradation, attenuate protein synthesis, and dampen inflammation and immunity [46,47]. Alpha-amylase activates the sympathetic nervous system [45] and exhibits diurnal variations, with its level in saliva being more sensitive to exercise-induced stress than that of cortisol [43]. Alpha-amylase also contributes to innate mucosal immunity [46,48,49].

### 4.4. Subjective Parameters

Assessment of subjective psycho-emotional variables [2], including self-reported sleep [50], perceived exertion [51] and general well-being, are crucial components of the monitoring of recovery and stress [11,52]. Although subjective indicators tend to be more sensitive to acute and chronic training loads than objective ones [11], the former can be more easily manipulated to achieve the outcomes desired.

Apps can be programmed to use touch or voice-controlled user-interfaces to monitor various subjective variables in a convenient manner.

### 4.5. Neuromuscular Variables

Applied properly [8], Wearables can detect neuronal activation of muscles that reflects neuromuscular fatigue [53]. POCT can be employed to measure blood levels of, e.g., aspartate aminotransferase, lactate dehydrogenase, creatine kinase, and myoglobin, classical markers of muscular load [54,55], with elevated levels indicating muscle damage and rhabdomyolysis. Since the kinetics of these levels vary, these parameters should be assessed in conjunction with the external load.

### 4.6. Parameters Related to Metabolism

Wearables can measure muscle oxygenation [8], thereby providing an estimate of local oxygen delivery and/or providing insights into muscular responses to exercise [56]. POCT can be utilized to measure blood levels of urea, uric acid and creatinine as reflections of metabolic processes. Urea in the capillary blood is indicative of augmented protein catabolism and gluconeogenesis [57,58]. An elevated level of uric acid, the terminal product of purine metabolism, is indicative of enhanced metabolism when muscle stores of glycogen have been depleted [55,57]. Creatinine levels provide information concerning renal functioning, which is of particular interest in situations where a proper electrolyte balance is crucial [55]. Levels of lactate can be monitored easily by both POCT devices and Wearables [59] and different lactate thresholds have been utilized as estimates of endurance performance [60].

POCT devices can also measure the partial pressures of oxygen and carbon dioxide in, as well as the pH of the blood, all important load variables e.g., in connection with hypoxic exposure [43].

POCT-assessed quantification of testosterone in the saliva may allow assessment of non-functional overtraining [46]. Moreover, the ratio of testosterone to cortisol provides further insight into the metabolic state (i.e., catabolic or anabolic) [43,61].

## 5. Practical Procedure for Monitoring Relevant Parameters

**Figure 1:**
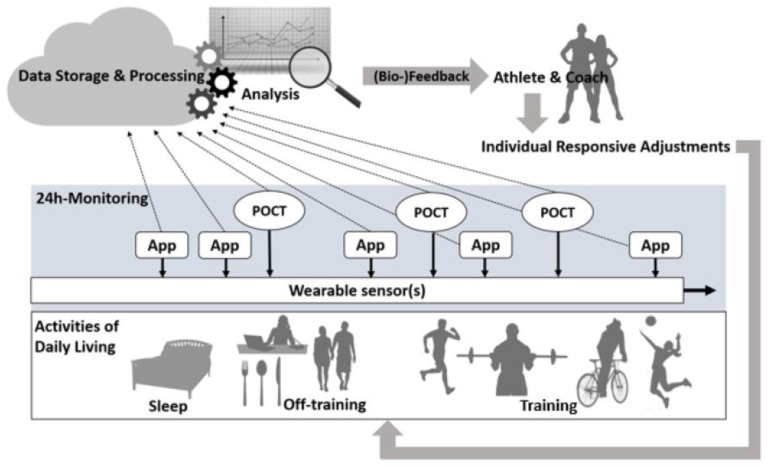
Procedure for monitoring external and internal training load employing Apps, Wearables and POCT and providing feedback to athletes and their coaches that allows beneficial responsive modification of exercise programs.

Figure 1 illustrates a procedure for individualized management of load and recovery designed to optimize performance and/or minimize the risk of overuse, injury and/or illnesses.

Wearables allow unobtrusive and continuous monitoring of parameters during training, recovery and periods of off-training [6], as well as, if approved by regulatory bodies [62], during actual competitions.

Apps that collect subjective data and require little compliance may be used selectively per day e.g., in the morning or before or after a training session. Since POCT requires sampling of capillary blood or saliva and the levels measured may show circadian variation, collection of such data daily or at shorter intervals might not be feasible. Since no individual App, Wearable or POCT device on its own can monitor all of the parameters mentioned above, in our opinion a combination of these devices is required in order to achieve a more holistic view of the various physiological, biomechanical and psychological responses of an athlete. It appears advantageous to incorporate at least wearable sensors into a body sensor network which is part of a fully integrated multiplexed sensing system [63], i.e., a central body unit connected wirelessly to various sensor nodes and cloud services that pre-format and synchronize relevant data [63,64], to facilitate user handling of several wearables at the same time.

The selection of parameters, as well as the timing and frequency of their monitoring clearly depends on the sport involved, the scientific basis for measurement, whether the individual is training or competing, and the extent to which the athlete and his/her coach accept and/or are aware of the benefits and drawbacks of monitoring with Apps, Wearables, and POCT.

It is beyond our present scope to discuss the numerous and rapidly changing technologies and algorithms involved in Apps, Wearables and/or POCT devices and, therefore, we refer practitioners to information concerning the advantages and disadvantages of each [8,65]. Currently, there are few reports involving 24-h monitoring in a sports setting [27,66] and more research in this area is clearly warranted.

As the amount of data collected increases, more effective systems for analysis, interpretation and reporting simple, yet meaningful results to athletes and coaches are necessary. With advancements in the analysis of large datasets, suitable algorithms may allow novel insights into the relationships between the parameters monitored and various aspects of performance and/or health. To further improve the framework outlined, we propose that future developments must allow the monitoring of additional parameters non-invasively by Wearables and/or Apps to collect as much data as reliable and as conveniently as possible. Moreover, an easy-to-use system that ideally incorporates all of the parameters mentioned above to provide simple, but powerful feedback to the practitioner is required.

Only if the data collected are stored securely to avoid misuse [67] can the framework outlined be employed successfully by stakeholders.

## 6. Practical Considerations

In this overview we have proposed a procedure for assessing markers of external and internal load by Wearables, Apps and/or POCT in a minimally or non-invasive manner designed to adapt training programs in order to optimize performance and/or minimize the risk of injury and/or illness. However, we have not taken certain other parameters usually monitored by other (invasive) procedures (e.g., venous blood sampling or muscle biopsies) into consideration. Furthermore, we have not discussed the methods and analytical algorithms involved extensively. Since different technologies and algorithms probably provide different results, we advise practitioners to carefully check the reliability and validity of each device of interest carefully following outlined recommendations prior to application in a routine monitoring [68,69] Moreover, when choosing a Wearable, App or POCT device for use, practical considerations such as the costs of the device(s) and of each measurement, as well as the time required for analysis, battery life, options for transfer of data and data security must be taken into consideration.

Finally, we want to emphasize that a framework as suggested here will vary depending on the technology employed, training status, sport and individual goals and most notably the framework does not replace coaching intelligence and the athlete’s experience but may assist to enhance performance and/or to reduce the risk of overuse, injury and/or illness.

## 7. Conclusions

Here, we summarize external and internal parameters that can be obtained by Apps, Wearables, and/or POCT and utilized to enhance athletic performance and/or reduce the likelihood of injury and/or illness; we also propose a procedure for such monitoring. For practical purposes, a sophisticated data management system will be required, as well as additional evaluation of the relationships between various parameters and performance and/or health.

## Figures and Tables

**Table 1:** Important external parameters and metrics that can be monitored by Apps and Wearables.

Type of Parameter	Individual Parameters	Method/Sensor Technology	Additional Comments
Duration and frequency of training sessions	- Time - Number	Sport watches	Sport watches allow automatic storage of data in the “cloud”
Distance covered (in different speed zones)	e.g., - absolute value - relative value - acute:chronic workload ratio	Global Navigation Satellite Systems	- Only useful outdoors - High sampling frequency required
		Local positioning systems	In- and outdoors
Short explosive activities	e.g., - absolute accelerations - relative accelerations	Inertial measurement units	Embedded in a Global Navigation Satellite System receiver unit
Sleep	- Quantity - Circadian rhythm	Actigraphy	Actigraphy should only be used with caution to access sleep quality.
Environmental factors	- Temperature - Altitude - Ultra-violet radiation - Humidity	- Thermometer - Barometer - Hygrometer	

**Table 2:** Important internal parameters and metrics that can be monitored by Apps, Wearables and point-of-care-testing.

Type of Parameter	Individual Parameter	Area of Interest
General health	Core, body or skin temperature	Thermoregulation
	White blood cell count	Infections
	High-sensitive C-reactive Protein	Inflammation
	Immunoglobulin A (IglA)	Mucosal immune function
	Reactive Oxygen Species	Oxidative stress
	Haemoglobin	Anaemia and dehydration
	Ferritin	Iron deficiency
Bio-psychological stress	Cortisol	- Protein degradation - Suppression of immune function
	Alpha-amylase	Stress on the sympathetic nervous system
Subjective parameters	Questionnaires and diaries	Various psychological aspects
Parameters of cardiac stress	Cardiac troponin	Myocardial stress
	Fatty acid-binding protein	
	Heart rate during exercise	
	Heart rate variability	Cardiac autonomous nervous system
	Heart rate recovery	Overreaching
Parameters of muscle damage	Aspartate aminotransferase	Breakdown of muscle cell structureProtein catabolism
	Creatine kinase	
	Myoglobin	
	Lactate dehydrogenase	
Parameters of metabolism	Lactate	Endurance performance
	Urea	Elevated protein catabolism
	Uric acid	Enhanced metabolic strain when muscle stores of glycogen are depleted
	Creatinine	Renal functioning
	Testosterone	Non-functional overreaching
	Tissue oxygenation	Intensity of effort
	pH	Acid-base status
